# Correlation between Melatonin and Colostral Regulatory T Cells in *Giardia lamblia* Infection

**DOI:** 10.3390/biom14070744

**Published:** 2024-06-24

**Authors:** Adriele Ataides de Queiroz, Eduardo Luzía França, Gabriella Regina Borges Gadenz, Letícia Damas Leão Dalcin, Mahmi Fujimori, Danielle Cristina Honorio França, Maria Aparecida Gomes, Adenilda Cristina Honorio-França

**Affiliations:** 1 Institute of Biological Sciences, Department of Parasitology, Federal University of Minas Gerais, Belo Horizonte 31270-901, Brazil; adriele.queiroz@ufmt.br (A.A.d.Q.); magomes@icb.ufmg.br (M.A.G.); 2 Institute of Biological and Health Sciences, Federal University of Mato Grosso, Barra do Garças 31270-901, Brazil; eduardo.franca@ufmt.br (E.L.F.); gabriellagadenz@gmail.com (G.R.B.G.); let_damas@hotmail.com (L.D.L.D.); mahmi_fujimori@yahoo.com.br (M.F.); daniellechfranca@gmail.com (D.C.H.F.)

**Keywords:** colostrum, giardiasis, hormone, cytokines, Treg cells

## Abstract

Giardiasis is a parasitic disease caused by *Giardia lamblia* (*G. lamblia*) that affects people worldwide. Still, few studies report on the immunoregulatory effects of the biomolecules of colostrum during interactions with *G. lamblia*. This study aimed to assess the concentrations of melatonin and cortisol hormones, the percentage of Treg cells, and the levels of cytokines IL-10 and TGF-β in colostrum from mothers who tested positive for the parasite. This cross-sectional study analyzed colostrum samples from 25 puerperal. The samples were tested using an ELISA to determine if they were seropositive for *G. lamblia* and the type of antibody present (IgM and IgG). Based on the results, the samples were divided into three groups: a control group (N = 10) with no reaction to either IgM or IgG, a group seropositive for IgG (IgG^+^/IgM^−^; N = 8), and a group seropositive for IgM (IgM^+^/IgG^−^; N = 7). The concentrations of melatonin and cortisol were measured using the ELISA method. Additionally, cytokines IL-10 and TGF-β and immunophenotyping were analyzed using flow cytometry. In the group that tested positive for IgM anti-*G. lamblia*, the concentration of melatonin was lower. However, in the colostrum from mothers who tested positive for IgG anti-*G. lamblia*, the level of this hormone had increased. The cortisol levels were similar between the groups, regardless of seropositivity. There was a higher percentage of Treg cells in the colostrum from mothers who tested positive for IgM anti-*G. lamblia*. TGF-β levels also increased in the colostrum of mothers who tested positive for IgM anti-*G. lamblia*. In the seronegative group for *G. lamblia*, there was a positive correlation between melatonin concentration and the percentage of Treg cells. These data suggest that the increase in regulatory cells and cytokines and the reduction in melatonin in colostrum from mothers with recent giardia infection may contribute to the evolution and manifestation of the disease.

## 1. Introduction

Giardiasis is caused by the protozoan *Giardia lamblia* (*G. lamblia*) [1] and affects people worldwide. Considered a neglected disease [2,3], it can be asymptomatic or present a series of symptoms of giardiasis, including abdominal colic, nausea, vomiting, bloating, acute or chronic diarrhea, and intestinal malabsorption, since the protozoan colonizes mainly the small intestine, resulting in damage to enterocytes and impaired epithelial barrier function [1,4]. These alterations cause changes in the absorption of nutrients along the intestine, causing malnutrition and anemia, especially in children [5]. Several studies aim to elucidate the differences between the variations of symptoms. It is believed that a diversity of factors can lead to the various forms of the clinical manifestations of the disease, including parasitic load [6], infection-associated giardiasis [7], antigenic variation in the parasite [8], the immune status of the host [9], and hormonal variations [10].

Immunocompetent hosts develop efficient immune responses to prevent or restrict parasitism [11]. However, this response can be self-limited in some cases [12], indicating the presence of mechanisms of the parasite itself or the host capable of interfering during the infection.

Giardiasis impacts pregnancy, becoming a debilitating disease that can compromise maternal and fetal well-being and breastfeeding [13]. Effective breastfeeding prevents parasitic infections like *G. lamblia*, as human colostrum macrophages can eliminate *G. lamblia* trophozoites in vitro [10,13,14].

Breast milk contains regulatory T cells (Tregs) essential for maintaining and inducing tolerance in newborns. These cells, representing 5% to 10% of CD4^+^ T cells, regulate immune responses to food, self-antigens, and microbiota. They are identified by their high expression of the IL-2 receptor α chain (IL-2R), also known as CD25, and the presence of the transcription factor FOXP3 (forkhead box protein 3), which indicates their regulatory function [15]. Colostrum also has bioactive components that act without causing inflammation, demonstrating that colostrum and mature milk have anti-inflammatory components [16]. Among these components, IL-10 and TGF-β are important immunomodulatory cytokines and stimulate intestinal maturation and the newborn’s defense [17]. Furthermore, during infections by *G. lamblia*, these cytokines prevent the recruitment of inflammatory cells, inducing an intestinal regulatory immune response [18]. In addition to cells and cytokines, some studies demonstrate the presence of the melatonin and cortisol hormones and the interaction with the cells present in colostrum [19,20,21] during interactions with *G. lamblia*, suggesting that both hormones can modulate the functional activity of these colostrum cells [10].

On the other hand, it considers the habitat of *G. lamblia* and the mucosa-associated lymphoid tissue (MALT), an environment with non-inflammatory characteristics that can be immunomodulated. Furthermore, the potential anti-inflammatory effects of melatonin and cortisol and the presence of intestinal regulatory T cells that produce large amounts of anti-inflammatory cytokines, such as IL-10 and TGF-β, are crucial in regulating immune responses and could potentially act together. Thus, variations between these components in secretion may be the key to understanding symptomatic *G. lamblia* infections [5,22]. The role of colostrum as a rich source of immunoregulatory components and hormones, which are crucial for stimulating the immune system of newborns and preventing diarrheal diseases, may be important. Therefore, it is essential to evaluate the impact of maternal giardiasis on these components. Additionally, it is necessary to establish the role of colostrum’s immunological factors in the G. lamblia infection process. Thus, this study analyzed the immunoregulatory components in the colostrum of seropositive mothers for *G. lamblia*.

## 2. Materials and Methods

### 2.1. Subjects

The cross-sectional study included nursing mothers who were seropositive or not for *G. lamblia*. Colostrum and blood samples were obtained from nursing mothers using the Public Health Service in Barra do Garças—MT. All mothers signed the Free and Informed Consent Form (ICF); the samples were collected after signing. The local ethics committee of Araguaia approved this study (Protocol number CAAE: 05524918.0.0000.5587).

### 2.2. Obtaining Serum and Determination of Serology for G. lamblia

Blood samples of 10 mL were collected from each mother between 48 and 72 h after postpartum. The samples were placed in tubes without anticoagulant and then centrifuged at 160× *g* for 15 min to separate the serum. The serum samples were stored at −80 °C to determine *G. lamblia* serology. 

Reactivity for *G. lamblia* was performed in serum using the ELISA method (enzyme-linked immunosorbent assay) to determine the reactivity of IgM and IgG antibodies to *G. lamblia*. The trophozoite cultures used were from the Portland-1 strain (ATCC^®^ 30888TM) in axenic culture TYI-S-33 medium [23]. The antigen preparation and plate sensitization steps were performed following adaptations of the method used by Pacheco [24]. The Bradford method determined the concentration of soluble proteins in the supernatant [25] and frozen at −20 °C until use. The reading was performed in a spectrophotometer at 490 nm. To determine the cut-off point, the ROC (receiver operating characteristic curve) was analyzed by MedCal^®^ software version 22.026, presenting sensitivity and specificity values of 100% and the area under the ROC (AUC—area under the curve) of 100%, where an OD (optical density) of 0.194 for IgM and 0.107 for IgG is the cut-off value of reactivity for *G. lamblia* [24].

A total of 43 mothers assisted in the Public Health Service were evaluated. According to the results, 25 pregnant women were included and distributed as follows: a seronegative group for *G. lamblia* (the control group) comprising mothers who tested negative for both antibodies (N = 10—IgM^−^/IgG^−^); an IgM anti-*G. lamblia* seropositive group comprising mothers who tested positive for IgM (N = 7—IgM^+^/IgG^−^), indicating recent infection; and a group seropositive for IgG anti-*G. lamblia*, comprising women who only showed positivity for IgG anti-*G. lamblia* antibodies (N = 8—IgM^−^/IgG^+^), indicating previous infection. Mothers who tested positive for both IgM and IgG anti-*G. lamblia* were excluded from the study.

The sample size proposed in this study was based on statistical calculations of the sample size assuming a loss to follow-up of around 10% and correcting for the effects of α (5%) and β (20%) errors attributed to the study.

**Figure 1:**
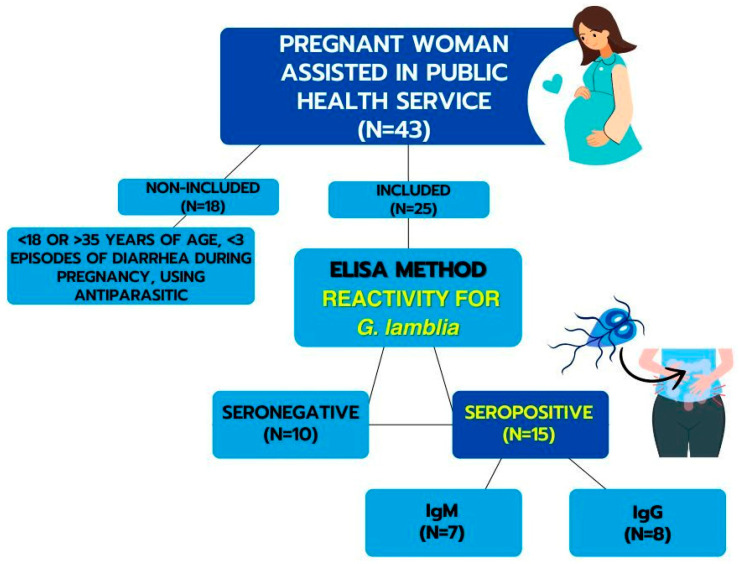
Representative scheme for obtaining samples.

The groups were controlled for several variables. The study included postpartum women between the ages of 18 and 35 who had experienced at least three episodes of diarrhea during pregnancy, gave birth to a healthy baby without any malformations, had a gestational age at delivery between 37 and 41^6/7^ weeks, and tested negative for hepatitis, HIV, and syphilis. None of the participants had used antiparasitic medication. The scheme for obtaining samples is shown in Figure 1.

### 2.3. Obtaining Supernatant and Colostrum Cells

Approximately 8 mL of colostrum was collected between 48 and 72 h postpartum. Colostrum samples were collected between 8:00 and 10:00 in the daytime. The colostrum was centrifuged for 10 min at 160× *g*, at 4 °C, and separated into three clear phases: the cell pellet, an intermediate aqueous phase (supernatant), and a lipid-containing supernatant. The upper fat layer was discarded, and the aqueous supernatant was stored at −80 °C for later measurement of antibodies (IgM and IgG) for anti-*G. lamblia*, melatonin, cortisol, IL-10, and TGF-β. The cell pellet was reserved for later analysis.

The colostrum cells were separated in density gradient by Ficoll-Paque (Pharmacia) for 40 min at 160× *g*, at 4 °C, producing preparations with 98% pure mononuclear cells, analyzed by light microscopy. Mononuclear cell concentrations were adjusted to 2 × 10^6^ cells/mL in a Newbauer chamber. Cell viability was assessed by Trypan blue exclusion. The proportion of non-viable cells was calculated based on the number of Trypan blue-stained (non-viable) cells compared to the total 100 cells, and the analysis resulted in 99% viable cells. The cells were used for the immunophenotyping assays.

### 2.4. Melatonin Concentration Dosage

Melatonin was extracted from colostrum supernatant and quantified by a commercial determined by an Immuno-Biological Laboratories ELISA kit (IBL, Hamburg) with the following characteristics: the lower detection limit was 1.6 pg/mL, and intra-assay and inter-assay coefficients of variation (%) were 3.0–11.4 and 6.4–19.3, respectively. The extraction of melatonin was performed using the affinity chromatography method with standardized columns. The columns were placed in glass tubes and centrifuged twice with 1 mL of methanol (1 min—200× *g*). Then, the columns were washed twice with double-distilled water (1 min—200× *g*). After preparing the columns, 0.5 mL of standards, controls, and samples were applied and centrifuged for 1 min at 200× *g*. After applying the samples and standards, the columns were washed again with 1.0 mL of 10% methanol for 1 min at 500× *g*. Next, the eluate containing the hormone melatonin was extracted by adding 1.0 mL of methanol at 200× *g*. After obtaining the eluate, the methanol was evaporated using an evaporator centrifuge (speed-vac). The material was reconstituted with 0.15 mL of double-distilled water under stirring for 1 min and immediately analyzed.

Fifty milliliters of each standard control colostrum were placed in an ELISA plate with 50 mL of melatonin-biotin and 50 mL of antiserum in each well. The plate was incubated at 4 °C for 20 h. After this period, the supernatant was discarded, the plate was washed three times with wash buffer, and 150 mL of the conjugated enzyme was added. After 120 min. of incubation at room temperature, the plate was washed three times, 200 mL of the substrate p-nitrophenyl phosphatase (PNPP) was added, and incubated for another 40 min under agitation. After this period, the reaction was blocked with 50 mL of “PNPP stop” solution. The reading was performed in a plate spectrophotometer at 405 nm. The results were obtained using a standard curve and expressed in pg/mL.

### 2.5. Cortisol Concentration Measurement

Cortisol in the colostrum supernatant was quantified by a commercial microplate ELISA test by Kit DRG^®^ International, Inc., Springfield, NJ, USA, with the following characteristics: the lower detection limit was 100 pg/mL, and intra-assay and inter-assay coefficients of variation (%) were 8.1 and 8.8, respectively. Amounts of 100 µL of each standard, control, and colostrum were placed in an ELISA plate with 200 µL of cortisol conjugated to peroxidase. The plate was incubated for 60 min at room temperature while shaking at 300 rpm. After this period, the supernatant was discarded, and the plate was washed five times with wash buffer. Subsequently, 200 µL of tetramethylbenzidine (TMB) substrate solution was added, and the plates were incubated for 30 min at room temperature. After this period, the reaction was blocked with 100 mL H_2_SO_4_ solution. The reading was performed in a plate spectrophotometer at 450 nm. The results were obtained using a standard curve and expressed in ng/mL.

### 2.6. Immunophenotyping

The colostrum cells were stained with 20 μL of anti-CD4-PE (BD Biosciences, San Jose, CA, USA), 20 μL of anti-CD25-FITC (Biosciences, San Jose, CA, USA), 20 μL of anti-CD4-PerCp (BD Biosciences), and 20 μL of Anti-FoxP3-PE (Biosciences, San Jose, CA, USA). The cells were incubated for 30 min at room temperature and protected from light. Then, the cells were washed and resuspended in phosphate-buffered saline [PBS] containing bovine serum albumin [BSA- Sigma, ST Louis, MO, USA; 5 mg/mL] for flow cytometry analyses. Before analyzing the samples, we verified the flow cytometer settings by following the manufacturer’s instructions and using tracking beads (BD Calibrite™ 3 Beads, BD Bioscience, San Jose, CA, USA). We then used compensation beads to establish compensation settings in FACSComp™ software on Mac^®^ OS 9 (BD Biosciences, San Jose, CA, USA). We applied the same compensation matrix to all samples to ensure high accuracy and reliability in our methodology.

**Figure 2:**
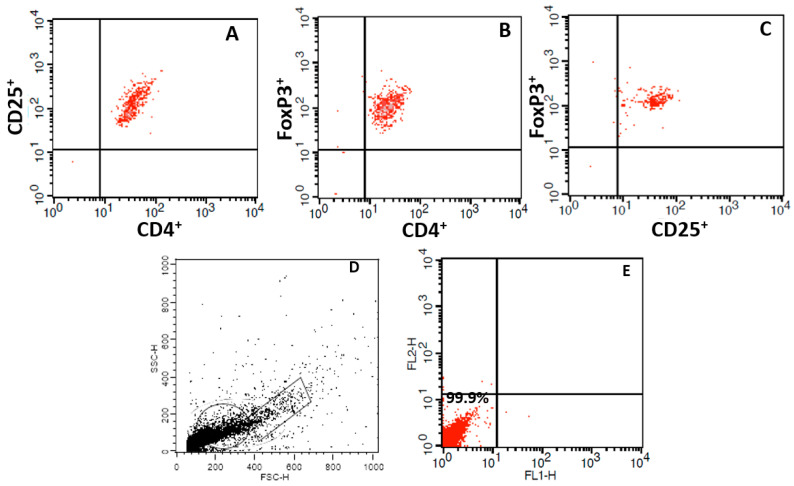
Flow cytometry dot plots of blood mononuclear cells labeled with specific surface markers. (**A**) represents cells that are double-positive for anti-CD4-PE and anti-CD25-FITC. (**B**) shows cells labeled with anti-CD4-PerCp and anti-FoxP3-PE. (**C**) displays cells that are positive for anti-CD25-FITC and anti-FoxP3-PE. Additionally, (**D**,**E**) depict the flow cytometry analysis of unlabeled colostrum mononuclear cells: the forward scatter (FSC), side scatter (SSC), and fluorescence (FL) channels.

A control was carried out with blood mononuclear cells and analyzed using a flow cytometer (Figure 2A–C). Isotypic controls (IgG1-FITC and IgG1-PE, both from BD Biosciences, San Jose, CA, USA) were also evaluated. Unlabeled colostrum cells were used to tune the forward scatter (FSC), side scatter (SSC), and fluorescence (FL) channels, allowing light emission parameters to be detected and the frequency of lymphocytes and monocytes in the preparation to be identified (Figure 2D,E). A total of 10,000 cells were analyzed for size (FSC), granularity (SSC), and fluorescence intensity (FL). This approach was crucial to ensuring the accuracy and reliability of our data, which were acquired using flow cytometry (FACSCalibur, BD Biosciences, San Jose, CA, USA) and Cell Quest software version 7.5.3. The data were analyzed using Flowjo 7.2.5 software, and the coefficient of variation (CV) was 4. Intracellular staining for Foxp3 was performed on cells obtained after sorting according to the manufacturer’s instructions (Biosciences, San Jose, CA, USA).

### 2.7. Quantification of IL-10 and TGF-β Cytokines

Colostrum supernatant was collected and stored at −80 °C before analysis. The flow cytometry method quantified cytokine concentrations in the supernatant. IL-10 was evaluated by the Cytometric Bead Array Human Inflammatory Cytokines Kit (CBA, BD Bioscience, USA). TGF-β was evaluated by the BD™ Cytometric Bead Array (CBA) Human TGF-β1 Single Plex Flex Set (CBA, BD Bioscience, USA) kit. The reading was performed using a flow cytometer (FACSCalibur, BD Bioscience, USA).

### 2.8. Statistical Analysis

The data were expressed as mean ± standard deviation (SD). Statistical analyses were performed with the BioEstat^®^ version 5.0 software [Mamirauá Institute, Belém, Brazil]. A D’Agostino normality test and analysis of variance (ANOVA) were used for the statistical analysis of the data, followed by Tukey’s means comparison test. In addition, the Spearman correlation test was used. The results were considered significant when the *p*-value was less than 0.05 (*p* < 0.05).

## 3. Results

The nursing mothers had a mean age in years of 24.3 ± 6 and a mean gestational age in weeks of 38.8 ± 0.7. IgM and IgG antibodies were examined in both serum and colostrum supernatant. All serum samples that tested positive for IgG anti-*G. lamblia* (IgM^−^/IgG^+^), and 90% of serum samples tested positive for IgM anti-*G. lamblia* (IgM^+^/IgG^−^) also showed reactivity in colostrum supernatant.

**Figure 3:**
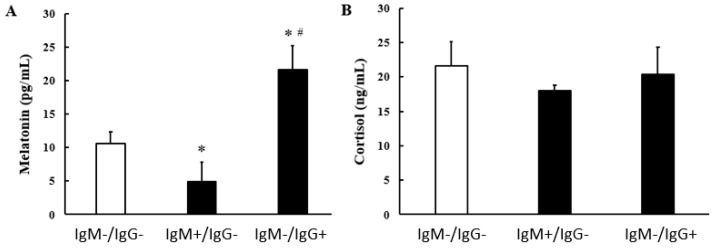
Melatonin concentration (pg/mL) (**A**) and cortisol concentration (ng/mL) (**B**) in colostrum supernatant from nursing mothers with seropositivity and seronegative for IgM and IgG Anti-*G. lamblia.* Data are expressed as mean and standard deviation (SD). * Indicates intergroup difference (IgM^−^/IgG^−^ and IgM^+^/IgG^−^ and IgM^−^/IgG^+^). # Indicates difference between IgM^+^/IgG^−^ and IgM^−^/IgG^+^ groups. *p* < 0.05.

Melatonin concentration was lower in the colostrum of *G. lamblia* IgM-seropositive mothers (Figure 3A). At the same time, the levels of this hormone were higher in the colostrum of mothers in the IgG anti g *lamblia* reagent group (Figure 2A). Regardless of positivity for *G. lamblia*, cortisol hormone concentrations were similar between the evaluated groups (Figure 3B).

**Figure 4:**
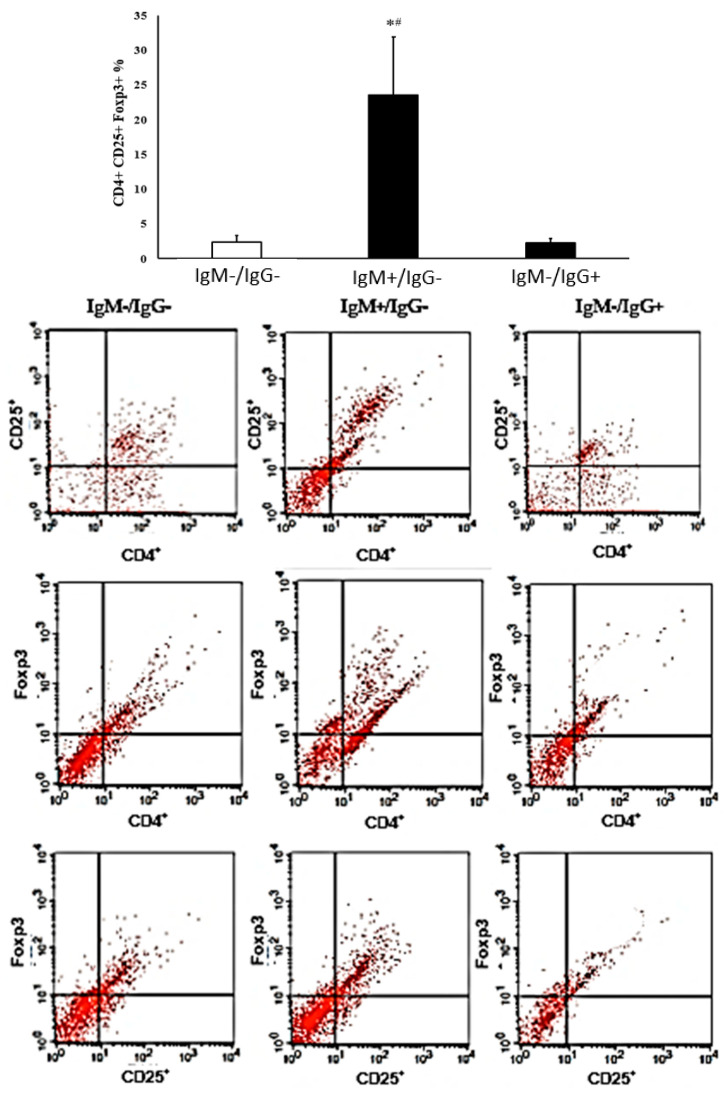
T cell surface phenotypes regulate mothers’ seropositive and seronegative colostrum for IgM and IgG anti-*G. lamblia*. Data are expressed as mean and standard deviation (SD). * Indicates intergroup difference (IgM^−^/IgG^−^ and IgM^+^/IgG^−^ and IgM^−^/IgG^+^). # Indicates difference between IgM^+^/IgG^−^ and IgM^−^/IgG^+^ groups. *p* < 0.05.

The percentage of regulatory T cells was higher in colostrum from IgM-reactive mothers compared to the control group. In the colostrum of mothers of the IgG anti-*G. lamblia*, the percentages of CD4^+^CD25^+^FOXP3^+^ cells were similar to those observed in the colostrum from non-reactive mothers (Figure 4).

IL-10 concentrations, regardless of reactivity to *G. lamblia*, were similar between the evaluated groups. However, the concentration of TGF-β increased in the group of mothers who were seropositive for IgM anti-*G. lamblia* (Table 1).

A negative correlation was observed between melatonin concentration and the percentage of regulatory T cells in the colostrum of mothers seronegative and IgM seropositive for *G. lamblia* (Table 2). However, regardless of the reactivity to *G. lamblia*, there was no correlation between melatonin, cortisol, and the cytokines IL-10 and TGF-β present in the colostrum of the evaluated mothers.

## 4. Discussion

Giardiasis is a re-emerging parasitic disease with high rates of diarrheal diseases and a prevalence of 50.08% of cases in children aged 3 to 12 years, especially in emerging countries such as Brazil [26,27], where diarrheal diseases account for more than 20% of child deaths and the impact of these diseases is particularly severe. In contrast, this rate is less than 1% in more developed countries, indicating a significant disparity in healthcare outcomes [28,29]. 

In this context, breastfeeding becomes an important source of protection against giardiasis. Colostrum, the first milk produced by the mother, contains bioactive components for the control of *G. lamblia*, including cells, hormones, and cytokines [10]. Human colostrum also contains natural components such as bile salt-stimulated lipase (BSL), which plays a key role in fighting parasites by breaking down the plasma membrane and inducing self-destruction [30]. This work shows that in infection by *G. lamblia*, changes occur in the concentration of the hormone melatonin, TGF-β, and the expression of Treg cells present in colostrum.

In parasitic infections, melatonin is an important factor in inducing an effector immune response and preventing tissue damage caused by infections with protozoa and helminths [31,32,33]. A study showed that melatonin reduced the number of blood trypomastigotes in rats infected with *Trypanosoma cruzi* during acute infections. It also decreased leukocytes, IL-2, TNF-α, and IFN-γ at the peak of parasitemia. Additionally, melatonin protects cardiac tissue by reducing inflammatory infiltrates and the presence of amastigotes at the site [34].

During infections by *G. lamblia*, an increase in the concentration of the hormone melatonin was observed [35], which could be explained by its immunomodulatory action, which is important in the control of giardiasis, considering that this same hormone is capable of increasing the phagocytosis of the mononuclear phagocytes in colostrum in contact with *G. lamblia* trophozoite [10,13]. In the present study, melatonin concentration decreased in the colostrum from mothers who had recent contact with *G. lamblia*. At the same time, this hormone was increased in the colostrum from seropositive mothers for IgG, suggesting that the parasite may interfere with melatonin production and favor the infection process in a recent infection. 

Melatonin and cortisol have been shown to regulate the functional activity of colostrum cells during interaction with *G. lamblia*, and they are important defense mechanisms for the protection and treatment of parasitic diseases [10]. No significant changes were observed in colostrum cortisol levels between the studied groups, regardless of *G. lamblia* seropositivity. A study on gerbils infected with *G. lamblia* and treated with dexamethasone demonstrated that this glucocorticoid could inhibit the formation of inflammation points in the duodenal epithelium. It also helped maintain long and intact villi and promoted an increase in the area of intestinal mucins. These findings suggest that the drug was responsible for preventing the appearance of lesions during enteritis caused by *G. lamblia* infection [36].

Interestingly, in the colostrum from mothers with recent *G. lamblia* infection, there was an increase in CD4^+^CD25^+^FOXP3^+^ cells and an increase in TGF-β levels, suggesting that *G. lamblia* infection favors a regulatory environment and that it can probably be a determinant for symptomatic infections since these mothers had recent diarrhea. Treg cells are cell populations capable of inducing immunological tolerance, making them important in maintaining the homeostasis of the immune system [37].

It is known that intestinal Treg cells can regulate immune responses to food, autoantigens, and microbiota by secreting anti-inflammatory cytokines, such as IL-10 and TGF-β [38]. Children parasitized with *G. lamblia* showed a significant increase in the levels of natural regulatory T cells [39]. The presence of these cells during giardiasis is crucial, as they help to balance the immune response, thus preventing tissue damage. Furthermore, controlling the immune response is also important due to virulent components in the parasite, including the G. lamblia arginine deiminase enzyme (GlADI). This enzyme triggers the activation of innate receptors such as Toll-like receptors (TLRs), specifically TLR-4. This activation leads to an inflammatory state characterized by the release of pro-inflammatory cytokines and a decrease in IL10 [40].

A study with cattle infected with *G. lamblia* showed that the proliferation of TCD4^+^FOXP3- cells occurs, suggesting the existence of regulatory mechanisms during giardiasis [41] that may contribute to the parasite’s evasion in the face of immune responses. On the other hand, in mice with susceptibility to infection by *G. lamblia*, there is an increase in the number of Treg cells to Th17, indicating a suppression of the inflammatory response driven by Th17 and hindering a protective response to the parasite [42].

It has been found that the hormone melatonin affects Treg cells. When melatonin is administered in vitro, it reduces the response of Th1 and Th17 cells and the production of related pro-inflammatory cytokines. In addition, melatonin increases FOXP3 expression and IL-10 production in CD4^+^ cells, which indicates that melatonin has an immunoregulatory action [43].

This study revealed a correlation between melatonin and regulatory T cells. Melatonin is a hormone associated with regulating CD4^+^CD25^+^ Tregs and their Foxp3 expression [44,45]. This hormone appears to have little effect on Treg cells in normal situations but significantly increases the number of these cells in inflammatory diseases. Melatonin can act as an anti-inflammatory agent in healthy individuals, reducing Th1 and innate responses, while in certain pathologies, it can increase the number of Treg cells that express FOXP3. Given the high variability of Treg cells, these effects are still the subject of intense investigation [46]. In our study, we observed a reduction in melatonin concentration and an increase in the percentage of Treg cells in mothers’ colostrum with a positive test for IgM anti-*G. lamblia*, suggesting that the inflammatory process caused by the parasite may have influenced the increase in these cells. It also indicates a potential link between susceptibility to parasitic infection, changes in Treg cell percentages, and changes in host microbiota composition [42].

CD4^+^CD25^+^FOXP3^+^ cells produce and release inhibitory cytokines, such as TGF-β [47]. Both humans and animals infected with *G. lamblia* produce various cytokines that contribute to eliminating the parasite [48]. It is known that IL-10 and TGF-β are produced at high levels in patients infected by protozoa, activating and modulating the immune cell response against these parasites [49,50] and that in the presence of *G. lamblia*, mononuclear cells increase their microbicidal activity when exposed to TGF-β [51].

This study showed an increase in TGF-β, but not in IL-10, in colostrum from mothers infected with *G. lamblia*. However, another study on mice infected with *G. lamblia* showed that the joint treatment of metronidazole and eucalyptol decreased inflammatory cytokines and increased IL-10 [52]. Similarly, a study on children infected with G. lamblia found an increase in IL-10, and it was found that this cytokine was linked to a reduction in airway inflammation [53]. Other studies on humans with *G. lamblia* infection have also shown increased IL-10 production and its significance in protecting tissues against excessive inflammatory responses [54,55,56].

A previous study also reports an increase in the production of TGF-β by mononuclear cells when exposed to trophozoites of *G. lamblia* [51]. A study comparing the production of various cytokines with different immune profiles and the generation of antibodies was conducted during experimental infection with G. lamblia and after oral immunization with Variant Surface Proteins (VSPs) in gerbils. The analysis revealed a significant increase in the production of TGF-β. This increase in TGF-β production was associated with increased levels of S-IgA (Secretory Immunoglobulin A) and a reduction in the incidence of intestinal inflammation in the immunized animals [57]. 

In general, TGF-β reduces the cellular immune response and, in some infections, determines symptomatic conditions [58]. In contrast, Tregs that release large concentrations of IL-10 prevent dysbiosis and intestinal inflammation in *G. lamblia* infection [59].

These cytokines have immunomodulatory activity in infection, limiting the inflammatory response. The release of IL-10 and TGF-β during infections by *G. lamblia* is important to prevent the recruitment of inflammatory cells [18]. The host itself can induce this intestinal immunoregulatory response to reduce inflammation.

The protection provided by breastfeeding does not depend solely on cells, hormonal levels, or other immunoreactive proteins. The amount, timing, and type of milk consumed, such as colostrum and mature milk, also play a crucial role [60,61]. This multifaceted nature of the protective effects of breastfeeding, which may vary depending on the type of milk, highlights the complexity of secretion.

This study focused mainly on the immunoreactive components of colostrum. Importantly, these components are also found in mature milk, suggesting that the protective effects of breastfeeding may extend to older breastfed children (10 to 12 months), thus offering long-term benefits [60,62]. This corroborates the importance of breastfeeding for child health and the need for additional research that delves into the different stages of milk maturation in mothers with *G. lamblia* infection.

The hormone melatonin, cells, and cytokines in colostrum play a significant role in secretion. These components may interact with other substances in secretion, suggesting that the protective effect of colostrum may result from a combination of specific and nonspecific immunological factors [10,63].

## 5. Conclusions

Mothers who have tested positive for the *Giardia lamblia* protozoan’s IgM serology have shown lower levels of the melatonin hormone in their colostrum. Additionally, they have an increased expression of regulatory T cells and concentration of TGF-β. Also, mothers who tested positive for IgG serology for *G. lamblia* showed increased melatonin hormone levels in their colostrum. These findings indicate that the changes in regulatory cells, cytokines, and melatonin hormone in the colostrum of mothers with recent giardiasis could potentially contribute to the disease’s progression and manifestation and may also be linked to the symptomatic infection.

## Figures and Tables

**Table 1:** IL-10 and TGF-β concentrations present in the colostrum of mothers seropositive (IgM or IgG) for *G. lamblia*.

Cytokines	Antibody Anti-*G. lamblia*		
	Non-Reagent	IgM^+^/IgG^−^	IgM^−^/IgG^+^
IL-10 (pg/mL)	25.53 ± 11.40	38.59 ± 7.17	32.47 ± 13.45
TGF-β (pg/mL)	95.73 ± 18.91	124.14 ± 2.14 *	103.05 ± 9.63

Data are expressed as mean and standard deviation (SD). * Indicates intergroup difference (IgM^+^/IgG^−^ and IgM^−^/IgG^+^). *p* < 0.05.

**Table 2:** Correlation between the hormone melatonin and cortisol concentration, percentage of Treg cells, and cytokines IL-10 and TGF-β.

Antibody Anti-*G. lamblia*	Non-Reagent		IgM^+^/IgG^−^		IgM^−^/IgG^+^	
	Rs	*p*	Rs	*p*	Rs	*p*
Melatonin/Cortisol	−0.2985	0.5655	0.6455	0.2394	−0.7537	0.0835
Melatonin/Treg	−0.8317	0.0401 *	0.8660	0.0476 *	−0.3339	0.5177
Melatonin/IL-10	−0.5643	0.3217	0.2962	0.6285	−0.2571	0.6228
Melatonin/TGF-β	0.2899	0.5773	0.5774	0.3080	0.4857	0.3287

* significant difference.

## Data Availability

The datasets generated during and/or analyzed during the current study are available from the corresponding author on reasonable request, without undue reservation.
